# The Role of Ovalbumin in Manganese Homeostasis during Chick Embryogenesis: An EPR Spectroscopic Study

**DOI:** 10.3390/molecules29133221

**Published:** 2024-07-07

**Authors:** Ana Vesković, Aleksandra M. Bondžić, Ana Popović Bijelić

**Affiliations:** 1 Faculty of Physical Chemistry, University of Belgrade, Studentski trg 12–16, 11158 Belgrade, Serbia; ana.veskovic@ffh.bg.ac.rs; 2 Vinča Institute of Nuclear Sciences, National Institute of the Republic of Serbia, University of Belgrade, P.O. Box 522, 11000 Belgrade, Serbia; aleksandrab@vin.bg.ac.rs

**Keywords:** EPR spectroscopy, manganese, metal binding, ovalbumin

## Abstract

Ovalbumin (OVA), a protein vital for chick embryo nutrition, hydration, and antimicrobial protection, together with other egg-white proteins, migrates to the amniotic fluid and is orally absorbed by the embryo during embryogenesis. Recently, it has been shown that for optimal eggshell quality, the hen diet can be supplemented with manganese. Although essential for embryonic development, manganese in excess causes neurotoxicity. This study investigates whether OVA may be involved in the regulation of manganese levels. The binding of Mn(II) to OVA was investigated using electron paramagnetic resonance (EPR) spectroscopy. The results show that OVA binds a maximum of two Mn(II) ions, one with slightly weaker affinity, even in a 10-fold excess, suggesting it may have a protective role from Mn(II) overload. It seems that the binding of Mn(II), or the presence of excess Mn(II), does not affect OVA’s tertiary structure, as evidenced from fluorescence and UV/vis measurements. Comparative analysis with bovine and human serum albumins revealed that they exhibit higher affinities for Mn(II) than OVA, most likely due to their essentially different physiological roles. These findings suggest that OVA does not play a role in the transport and storage of manganese; however, it may be involved in embryo protection from manganese-induced toxicity.

## 1. Introduction

Ovalbumin (OVA) constitutes over 50% of total hen-egg-white proteins, making it the predominant protein in the albumen. Additionally, OVA has been identified in other components of the egg, including the yolk, the vitelline membrane, and the eggshell [1]. Despite not exhibiting inhibitory activity towards serine and cysteine proteases, this globular glycoprotein of ~45 kDa has been classified within the serpin superfamily [1,2]. OVA is composed of 385 amino acids, including six cysteines, of which only two form a single disulfide bond. The determined crystal structure has revealed that almost all of the polypeptide chain forms a defined secondary structure, a mixture of α-helices and β-sheets [3,4]. OVA has been shown to be heterogeneous, both with respect to its covalently linked carbohydrate chain [5,6] and to the extent of phosphorylation of the two serine side chains [7]. Moreover, native OVA is mostly found in its dephosphorylated form [8]. In addition to its antioxidant properties due to the presence of free sulfhydryl groups, OVA also exhibits anticancer, antihypertensive, antimicrobial, and immune-modulating activities [2,9]. These properties have been recognized as advantageous for its various biomedical applications. Due to its well-documented immunogenic efficacy, it has been extensively utilized as a model protein antigen in vaccine development [10]. OVA-based nanoparticles have been shown as effective carriers for epigallocatechin-3-gallate in the treatment of ulcerative colitis [11]. OVA-coated Fe_3_O_4_ nanoparticles have exhibited promising results as reservoirs for chlorogenic acid, enhancing its anticancer effectiveness in vitro [12]. Recently, the Zn(II)/ZnO-OVO hybrid nanocomplex has demonstrated excellent antibacterial and antifungal activity, with the potential for application in antimicrobial formulations [13]. Certainly, these findings reveal that metal complexes/conjugates of OVA may find significant applications in biosensing, drug delivery, and diagnostics. In this context, a new type of fluorescent OVA-modified Au nanocluster (OVA-AuNC) sensor has been developed for the detection of ascorbic acid [14]. In another study, fluorescent OVA-AuNCs were shown to detect salicylaldehyde [15]; moreover, the salicylaldehyde/OVA-AuNCs nano-assembly was found to be a highly selective and sensitive probe for the detection of Hg²⁺ and folic acid [15]. Finally, it should be mentioned that OVA has also found extensive uses in the production of functional foods and pharmaceuticals due to its unique foaming, gelling, and emulsifying characteristics [16,17].

Despite being the first protein isolated in pure form from egg white, its actual physiological function is not fully elucidated. Found in its dephosphorylated form in egg yolk, OVA is proposed to mainly serve as a source of amino acids, supporting embryo growth and development [4]. A recent study has investigated if OVA, like serum albumins, may participate in fatty acid (FA) transport; however, it was concluded that OVA lacks a specific FA binding site [18,19]. The potential role of OVA in the transport and storage of metal ions has also been assessed [20]. A polarographic study has indicated the binding of Cu^2+^ and Cd^2+^ to the carboxyl and imidazole groups of the protein [21]. A single strong affinity binding site for various divalent and trivalent cations, including Mn^2+^, Co^2+^, Cu^2+^, Zn^2+^, Gd^3+^, and Dy^3+^, was detected by magnetic resonance water proton relaxation rate enhancements. Additionally, the presence of secondary, low-affinity binding sites was suggested [22].

In vivo, OVA presumably has a vital role in embryo nutrition, hydration, and antimicrobial protection. It has been shown that OVA migrates into the yolk and, subsequently, through the yolk sac membrane into the embryo [23]. Namely, starting from the 11th day of incubation, the egg-white proteins enter the amniotic fluid to be orally absorbed by the embryo [1,23]. The yolk contains essential minerals for the developing embryo, including manganese, iron, zinc, copper, calcium, and phosphorous [24]. The reported average contents of minerals (in mg per 100 g) of raw egg yolk and egg white, respectively, are as follows: Mn (0.055 and 0.011), Fe (2.73 and 0.08), Zn (2.30 and 0.03), Cu (0.077 and 0.023), Ca (129 and 7), and P (390 and 15) [25]. Manganese, as a cofactor for key metabolic and antioxidant enzymes, is vital for growth and development; its deficiency in humans and animals can lead to seizures, growth retardation, skeletal defects, diminished reproductive function, abnormal glucose tolerance, and altered metabolism [26,27]. It has also been found that manganese, besides calcium and vitamin D, is involved in maintaining optimal eggshell quality by promoting mucopolysaccharide synthesis. In this regard, studies have shown that supplemental manganese enhances thickness and fracture resistance, thus reducing damaged eggs [28]. To minimize economic losses, various strategies are currently implemented to enhance egg quality, with a primary focus on improving eggshell physical properties [29,30]. Namely, manganese is added to the hen diet, whether as sulfate or in inorganic form; however, excessive manganese exposure can cause neurotoxicity, leading to a Parkinson’s disease-like condition called manganism [26]. The normal mitochondrial function may be disrupted by manganese overload through mitochondrial ROS increase, inhibition of ATP production, and membrane permeability alteration, further causing mitochondrial dysfunction and eventually metabolic syndrome or other metabolic diseases [27]. Therefore, it is important to elucidate if OVA may be involved in the regulation of manganese levels in the egg, since this protein has been identified in multiple embryo organs and proposed to constitute a dynamic participant in organ cell development [23]. Moreover, as exposure to manganese in humans primarily occurs through food consumption in addition to skin absorption [31], it is imperative to know if a manganese-supplemented hen diet may possibly lead to high manganese content in eggs, particularly bound to OVA.

In this study, the binding of Mn(II) to OVA was investigated using X-band EPR spectroscopy. In addition, a specific graphical representation of the results is proposed, from which it is possible to obtain the total number of Mn(II) binding sites on any protein and distinguish between strong and weak binding sites. UV/vis and fluorescence spectroscopies were used to inspect possible changes in the OVA tertiary structure upon Mn(II) binding. Subsequently, Mn(II) binding was investigated for two other proteins, bovine serum albumin (BSA) and human serum albumin (HSA), and compared to OVA. These proteins were chosen due to their ability to bind and transport hundreds of endogenous and exogenous compounds, including metal cations. The aim of this study was to gain a better understanding of OVA’s role in the storage and regulation of manganese levels in vivo.

## 2. Results and Discussion

### 2.1. EPR Spectroscopy

#### 2.1.1. The Binding of Mn(II) to OVA

EPR spectroscopy can be used to investigate systems with unpaired electrons, such as free radicals and paramagnetic metals. Due to their extremely short spin–lattice (T_1_) relaxation times, EPR spectroscopy of metals is usually performed below 77 K since above this temperature the signals are broadened beyond detection. Namely, the lowering of the temperature slows down the spin–lattice relaxation, which increases the lifetime of the molecule in the excited state and, consequently, reduces the homogenous line-width (according to the Heisenberg’s uncertainty relation, the line-width is inversely proportional to the lifetime of the excited state) [32]. The low-temperature EPR spectroscopy provides essential information on the binding of metal ions to proteins and other biomolecules, such as their oxidation state, coordination environment and symmetry, and stoichiometry. Manganoproteins have been extensively studied by low-temperature EPR spectroscopy with the aim to characterize their active sites [33]. Since the samples for low-temperature EPR measurements are frozen, it is even possible to gain insight into the structure of short-lived intermediates containing Mn(III) or Mn(IV) oxidation states formed during protein–substrate catalytic reactions. Nevertheless, EPR spectroscopic studies of Mn(II) are also possible at room temperature due to the relatively long T_1_ relaxation time of Mn(II) compared to other metals [34]. Mn(II) shows an isotropic EPR sextet, i.e., the signal is split into six equal lines due to the hyperfine coupling of the unpaired electrons with the ^55^Mn nuclear spin (*I* = 5/2) [35]; however, this signal can arise only from the uncoordinated, free Mn(II) in solution—specifically the divalent hexaquo–manganous complex [Mn(H_2_O)_6_]^2+^—and not from the protein-bound cation that can be seen by low-temperature EPR spectroscopy [36,37,38,39]. Namely, it has been suggested that upon Mn(II) binding to proteins, the rotational freedom of Mn(II) becomes restricted due to the tumbling rate of the protein molecule, which is responsible for the apparent disappearance of the room-temperature EPR signal. In this respect, room-temperature EPR represents a highly sensitive spectroscopic technique for the study of manganese binding to proteins as it provides a quantitative assessment of the unbound Mn(II), from which the concentration of the protein-bound Mn(II) can be deduced.

**Figure 1:**
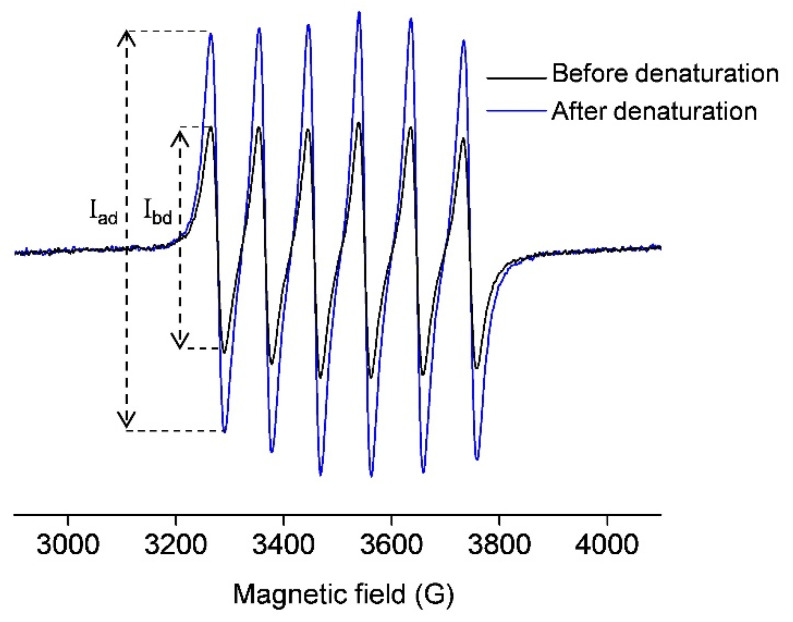
Room-temperature X-band EPR spectra of 0.5 mM OVA (in deionized water, pH 5.5) incubated with 1 mM MnCl_2_, before (black) and after (blue) denaturation with 1 mM perchloric acid (pH 3). The signals arise only from free Mn(II) in water ([Mn(H_2_O)_6_]^2+^).The intensities of the first lines in both spectra, from which the concentrations of Mn(II) were determined, are marked with I_bd_ and I_ad_ (before and after denaturation, respectively).

Figure 1 (black line) shows the room-temperature EPR spectrum of 0.5 mM OVA incubated with 1 mM MnCl_2_. This signal arises from the free Mn(II) in water ([Mn(H_2_O)_6_]^2+^) since the protein-bound Mn(II) does not exhibit a room-temperature EPR signal [36,37,38,39]. Upon OVA denaturation by 1 mM perchloric acid, the protein-bound manganese is liberated, and the EPR signal is accordingly increased (Figure 1, blue line). Again, this signal arises only from the unbound Mn(II). To quantitate the amount of free Mn(II) in both cases, the normalized intensity (the peak-to-peak amplitude) of the first line in the EPR spectra was measured (marked with I_bd_ and I_ad_ in Figure 1), and the concentration of Mn(II) was determined from the calibration plot for MnCl_2_ in deionized water (Figure S1 in Supplementary Materials). It should be pointed out that the quantitation of an EPR-active species using a calibration plot is possible here since the different signals have the same line-widths and line-shapes, and were measured under the same experimental conditions. The concentration of Mn(II) in the black spectrum was found to be 0.5 mM, and the concentration of Mn(II) in the blue spectrum was 1 mM. The first corresponds to the unbound Mn(II) concentration, [Mn_ub_], while the latter corresponds to the total concentration of Mn(II) initially added to OVA [Mn_tot_]; therefore, the concentration of the protein-bound Mn(II), [Mn_b_], can be obtained as the difference of the two, [Mn_b_] = [Mn_tot_] − [Mn_ub_], here equal to 0.5 mM.

**Figure 2:**
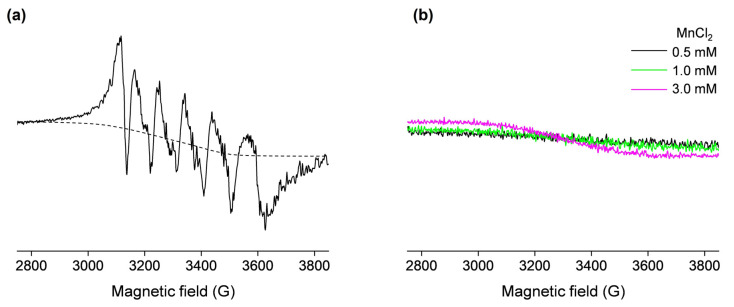
The 77 K X-band EPR spectra of (**a**) 0.5 mM OVA (in deionized water, pH 5.5) incubated with 1 mM MnCl_2_ (the dashed line shows the approximated background signal that arises from the unbound Mn(II)); (**b**) 0.5, 1, and 3 mM MnCl_2_ in deionized water (black, green, and magenta lines, respectively). The spectra in (**a**) and (**b**) are shown on the same scale.

In order to confirm the presence of the OVA-bound Mn(II) in the non-denatured sample containing 0.5 mM OVA and 1 mM MnCl_2_, the EPR spectrum was measured at 77 K (Figure 2a). This six-line signal, centered at *g* = 2, displays five higher-field hyperfine (forbidden) transitions caused by second-order effects due to cross products of the electron and nuclear spin operators and is characteristic of protein-bound Mn(II) ions [35,40,41]. Moreover, the spectrum shown in Figure 2a also contains a background signal (approximated with the dashed line), which arises from the unbound Mn(II) in water. Specifically, as observed from Figure 2b, free Mn(II) in water exhibits a very broad low-intensity signal at 77 K. The slope of this signal is increased with increasing Mn(II) concentrations, confirming it arises from a paramagnetic species. In general, the intensity and hyperfine couplings in Mn(II) EPR spectra are extremely sensitive to the nature of the ligands, and depend on the strength and geometry of the metal–ligand coordination bonds [35,42]; therefore, the shape and intensity of the signal shown in Figure 2b is the result of the very weak coordination of Mn(II) by water molecules in the [Mn(H_2_O)_6_]^2+^ complex.

Finally, it should be pointed out that, in principle, the amount of the protein-bound Mn(II) could be determined by double integration of the EPR signal measured at 77 K [43], shown in Figure 2a; however, this would require prior signal deconvolution and baseline correction. Also, the calibration plot could not be obtained for MnCl_2_ but rather a standard solution of 1 mM CuSO_4_ in 50 mM EDTA would need to be used [34,44]. Therefore, it seems reasonable that for the purpose of this study, the methodology for the determination of Mn(II) binding to OVA should involve the use of room-temperature EPR spectroscopy, rather than “the more demanding” low-temperature EPR. 

However, the low-temperature EPR spectrum shown in Figure 2a contains valuable information regarding the structural environment of the Mn(II) binding site on OVA, which can be obtained by measuring the hyperfine coupling constants A_hfc_ (i.e., the distances between hyperfine peaks in the spectrum). The spectrum exhibits an A_hfc_-strain (distribution in A_hfc_ values), which is most likely caused by the existence of slightly different protein conformations in the sample [34]. The determined average value of A_hfc_ was 96 G (260 MHz), suggesting that Mn(II) is octahedrally coordinated to oxygen ligands [35]. Indeed, it has been suggested that the carboxylate side chains from aspartate and glutamate near the N-terminal of OVA may act as Mn(II) ligands [22]. The same study has proposed that the primary ligand for Mn(II) has a pKa > 8, pointing to tyrosine and lysine side chains.

#### 2.1.2. The Determination of the Total Number of Mn(II) Binding Sites on OVA

**Figure 3:**
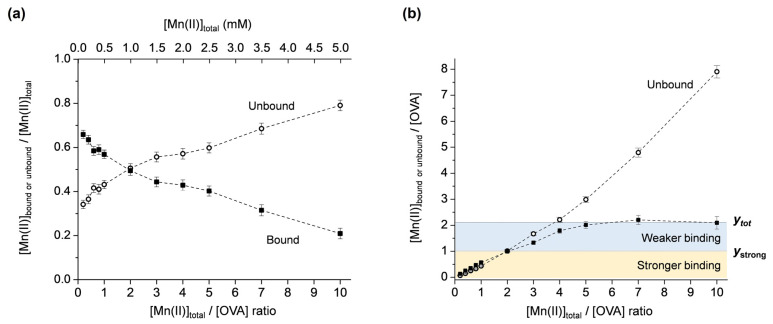
(**a**) Molar fractions of the unbound and bound Mn(II) ([Mn_ub_]/[Mn_tot_] and [Mn_b_]/[Mn_tot_]) vs. [Mn_tot_]/[OVA] ratio. (**b**) Unbound and bound Mn(II):protein ratios ([Mn_ub_]/[OVA] and [Mn_b_]/[OVA]) vs. [Mn_tot_]/[OVA] ratio. The yellow and blue regions correspond to stronger and weaker Mn(II) binding to OVA; *y*_tot_ and *y*_strong_ denote the total number and the number of strong Mn(II) binding sites to OVA, respectively.

Mn(II) binding to OVA was investigated for the metal:protein ratios in the range of 0.2–10, maintaining the concentration of OVA constant (0.5 mM). The concentrations of unbound Mn(II), [Mn_ub_], were determined from the intensities of the first line in the room-temperature EPR spectra (Figure S2 in Supplementary Materials), as described above. The protein-bound Mn(II) concentrations, [Mn_b_], were obtained from [Mn_b_] = [Mn_tot_] − [Mn_ub_]. Next, the molar fractions of the unbound and bound Mn(II) ([Mn_ub_]/[Mn_tot_] and [Mn_b_]/[Mn_tot_]) were plotted as the function of the total Mn(II):protein ratio, [Mn_tot_]/[OVA], Figure 3a. Note that the data points are simply connected by dashed lines only for clarity of observation and not fitted with a specific function. It is observed that at [Mn_tot_]/[OVA] ratios < 2, the amount of [Mn_b_] is greater than [Mn_ub_], and at [Mn_tot_]/[OVA] ratios > 2, the amount of [Mn_b_] is smaller than [Mn_ub_]. For [Mn_tot_]/[OVA] = 2, the dashed lines intersect, and [Mn_ub_] = [Mn_b_]. This shows that for [Mn_tot_] = 1 mM, [Mn_ub_] = [Mn_b_] = 0.5 mM, confirming the result obtained from the protein-denaturation experiment (Figure 1).

In order to determine the total number of Mn(II) binding sites on OVA, the unbound and bound Mn(II):protein ratios ([Mn_ub_]/[OVA] and [Mn_b_]/[OVA]) were plotted vs. the total Mn(II):protein ratio, [Mn_tot_]/[OVA], Figure 3b. Note that [Mn_b_]/[OVA] represents the fraction of the protein with bound Mn(II); therefore, it defines the number of bound Mn(II) per one molecule of OVA. From the plot in Figure 3b, it is observed that the amount of [Mn_b_]/[OVA] (black squares) is almost linearly increased up to [Mn_tot_]/[OVA] = 2. For [Mn_tot_]/[OVA] > 2, a saturation of [Mn_b_]/[OVA] occurs, and finally, a plateau is reached at [Mn_tot_]/[OVA] = 5 and maintained up to [Mn_tot_]/[OVA] = 10. This implies that there are at least two types of Mn(II) binding. The first occurs up to *y* = 1 ([Mn_ub_]/[OVA] = [Mn_b_]/[OVA] = 1) (Figure 3b, yellow region), where the protein-bound Mn(II) concentration linearly increases and exceeds the concentration of the unbound Mn(II). The second occurs up to *y* = 2 (Figure 3b, blue region), where the unbound Mn(II) starts to dominate, while the protein-bound Mn(II) concentration starts to saturate and finally reaches a plateau for [Mn_tot_]/[OVA] = 5. These two regions are likely to correspond to stronger and weaker Mn(II) binding to OVA, respectively, as will be discussed further.

The graphical representation of the results in Figure 3b allows for a straightforward determination of the total number of Mn(II) binding sites on OVA, directly from the *y*-axis value that corresponds to the [Mn_b_]/[OVA] curve plateau, here abbreviated as *y*_tot_. The number of strong binding sites can be determined from the *y*-axis value that corresponds to the point of intersection of the [Mn_b_]/[OVA] and [Mn_ub_]/[OVA] curves, here abbreviated as *y*_strong_. Namely, as discussed above for Figure 3a, at the curve intersection point, the molar fractions of the bound and unbound Mn(II) are equal (=0.5). Before this point [Mn_b_]/[Mn_tot_] > [Mn_ub_]/[Mn_tot_], and after this point [Mn_b_]/[Mn_tot_] < [Mn_ub_]/[Mn_tot_]. This suggests that OVA displays two different affinities for Mn(II) binding before and after the point of intersection. The higher affinity is displayed up to *y*_strong_ and the lower affinity up to *y*_tot_. Therefore, it may be concluded that OVA has a total of two Mn(II) binding sites (*y*_tot_ = 2), of which one is a stronger binding site (*y*_strong_ = 1). Moreover, OVA binds a maximum of two Mn(II) ions per protein molecule, even in a 10-fold excess of the metal. These results are in relatively good agreement with the NMR relaxometric study that has shown that native OVA possesses one strong binding site for Mn(II) (K_d_ ≈ 6 × 10^−4^ M); the alkaline phosphatase-treated OVA has one site with slightly weaker affinity (K_d_ ≈ 8.3 × 10^−4^ M); while the acid phosphatase-treated OVA has two equivalent sites with much weaker affinities (K_d_ ≈ 1.3 × 10^−3^ M) [22]. It should be highlighted here that currently there are no other data in the literature reporting on Mn(II)/OVA interactions. The dissociation constants determined in this work for the two non-equivalent binding sites [45] on OVA are K_d1_ = (5.0 ± 0.7) × 10^−4^ M and K_d2_ = (8.0 ± 1.1) × 10^−4^ M, implying a slightly weaker binding of the second Mn(II) ion to OVA. 

Typically, the total number of protein-binding sites for a specific ligand is determined from the *x*-axis intercept of the least-squares data fits in the Scatchard plot [46]. Scatchard analysis can also indicate if the protein contains sites with different affinities towards the ligand. However, it does not provide the exact number of stronger and weaker binding sites, which can be obtained from the plot ([Mn_ub_]/[OVA] and [Mn_b_]/[OVA]) vs. [Mn_tot_]/[OVA], Figure 3b) proposed in this study.

#### 2.1.3. The Binding of Mn(II) to BSA and HSA

**Figure 4:**
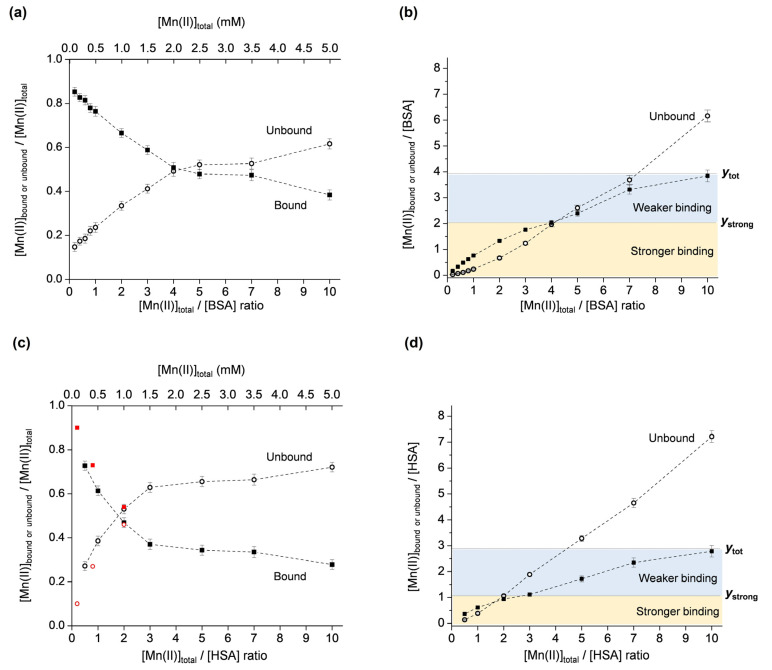
Molar fractions of the unbound and bound Mn(II) ([Mn_ub_]/[Mn_tot_] and [Mn_b_]/[Mn_tot_]) vs. [Mn_tot_]/[protein] ratio, for BSA (**a**) and HSA (**c**). Unbound and bound Mn(II)–protein ratios ([Mn_ub_]/[protein] and [Mn_b_]/[protein]) vs. [Mn_tot_]/[protein] ratio, for BSA (**b**) and HSA (**d**). The yellow and blue regions correspond to stronger and weaker Mn(II) binding to BSA or HSA, and *y*_tot_ and *y*_strong_ denote the total number, and the number of strong Mn(II) binding sites on BSA or HSA, respectively. The plot in (**c**) also shows data points in red taken from [49] obtained by ^1^H NMR relaxometry.

The described methodology was applied for the investigation of Mn(II) binding to two mammalian serum albumins, BSA and HSA. Both albumins were chosen since there is emerging evidence that although they exhibit high sequence similarity, their binding abilities are different [47,48]. As performed for OVA, the molar fractions of the unbound and bound Mn(II) were first plotted vs. the total Mn(II):protein ratio (Figure 4a for BSA and Figure 4c for HSA). It should be noted that the plot for HSA also contains data points (shown in red) that were taken from reference [49] for the defatted protein at pH 7, showing good agreement at low Mn(II):protein ratios. Subsequently, the second plot showing the unbound and bound Mn(II):protein ratios vs. the total Mn(II):protein ratio was constructed for both albumins (Figure 4b for BSA and Figure 4d for HSA). As proposed, the specific graphical representation of the results allows for the determination of the total number of binding sites on the protein, as well as the number of strong binding sites. The results indicate that, in the investigated Mn(II)-concentration range, BSA has a total of ~4 Mn(II) binding sites, of which two are stronger (*y*_tot_ = 3.85 and *y*_strong_ = 2, Figure 4b), and that HSA has a total of ~3 Mn(II) binding sites, of which one is stronger (*y*_tot_ = 2.80 and *y*_strong_ = 1, Figure 4d).

It is evident that in the investigated Mn(II):protein = 0.2–10 ratio range, Mn(II) binding to OVA is different than to HSA and BSA. OVA exhibits binding site saturation (observed as the plateau at *y* = 2 for ratios [Mn_tot_]/[OVA] = 5–10, in Figure 3b), while BSA and HSA do not display a plateau (Figure 4b and Figure 4d, respectively). It is, however, expected that the binding site saturation for BSA and HSA would occur for higher [Mn_tot_]/[SA] ratios, specifically greater than 10. The different binding modes of OVA and BSA/HSA are likely determined by their structures and physiological functions. The fact that even in a 10-fold excess of Mn(II), OVA does not bind more than two Mn(II) ions, may suggest that it does not play a role in the transport and storage of manganese. However, it may be involved in maintaining manganese homeostasis during early embryo development by preventing excessive Mn(II) concentrations in the albumen, and, subsequently, in the amniotic fluid and embryo. On the other hand, BSA and HSA are carrier proteins, transporting various substances such as fatty acids, bilirubin, hormones (e.g., thyroxine, cortisol), drugs, as well as metal ions or complexes through the bloodstream. Although albumins are not primary transporters of manganese, the interaction of manganese with BSA and HSA has been confirmed, primarily by NMR relaxometry. The available literature data are not in complete agreement (most probably due to different protein purities, pH, ionic strengths, temperatures, and sample incubation times), and here it is summarized chronologically for both proteins. In earlier studies, it was reported that BSA contains one strong (K_d_ ≈ 3.7 × 10^−5^ M) and five weak (K_d_ ≈ 3 × 10^−4^ M) binding sites [50], and that HSA contains two equivalent sites (K_d_ ≈ 6 × 10^−5^ M, or K_d_ ≈ 1.3 × 10^−4^ M for the defatted protein) for the binding of Mn(II) [51]. In another study using equilibrium dialysis, it was proposed that both HSA and BSA have approximately two strong binding sites for Mn(II), specifically, 1.8 sites for HSA (K_d1_ ≈ 8.3 × 10^−5^ M, K_d2_ ≈ 1.4 × 10^−4^ M), and 1.9 sites for BSA (K_d1_ ≈ 6.7 × 10^−5^ M, K_d2_ ≈ 1.3 × 10^−4^ M) [52]. Later, it was shown that HSA possesses one strong (K_d_ ≈ 1.3 × 10^−4^ M) binding site for Mn(II) ions per protein molecule, and several weaker binding sites [53]. These findings were based on the analysis of ^1^H T_1_ as the function of magnetic field strength, and of ^17^O T_2_ as the function of temperature, allowing the determination of various parameters involved in the relaxation process of the paramagnetic Mn(II)–HSA adduct. Furthermore, ^1^H NMR relaxometry has shown that Mn(II) binds to two independent sites on HSA (K_d1_ ≈ 1.1 × 10^−4^ M and K_d2_ ≈ 1.2 × 10^−3^ M), and that the primary binding site for Mn(II) corresponds to the secondary binding site for Zn(II), while the secondary binding site for Mn(II) corresponds to the primary binding site for Zn(II), specifically, the multimetal binding site A [49]. The binding of Mn(II) to the multimetal binding site A on HSA, with two inner-sphere water ligands that undergo rapid exchange, has later been confirmed by ^17^O NMR [54]. More recently, the interaction of Mn(II)/Mn(III) with BSA, investigated using fluorescence, UV/vis, and FTIR spectroscopies, has suggested moderate binding (K_d_ ≈ 1.4 × 10^−3^ M) of one manganese ion to the protein [31]. Finally, the results from this study, using EPR spectroscopy, show that HSA has one stronger (K_d_ = (1.8 ± 0.4) × 10^−4^ M) and two weaker (K_d_ = (2.6 ± 0.9) × 10^−3^ M) Mn(II) binding sites, and BSA has two stronger (K_d_ = (1.9 ± 0.2) × 10^−4^ M) and two weaker (K_d_ = (4.7 ± 1.5) × 10^−3^ M) Mn(II) binding sites, determined for globulin-free, fatty acid-free 0.5 mM protein preparations in deionized water (pH 5.5) in the 0.1–5 mM Mn(II) concentration range.

### 2.2. Fluorescence and UV/vis Absorption Spectra of OVA in Presence of Mn(II)

**Figure 5:**
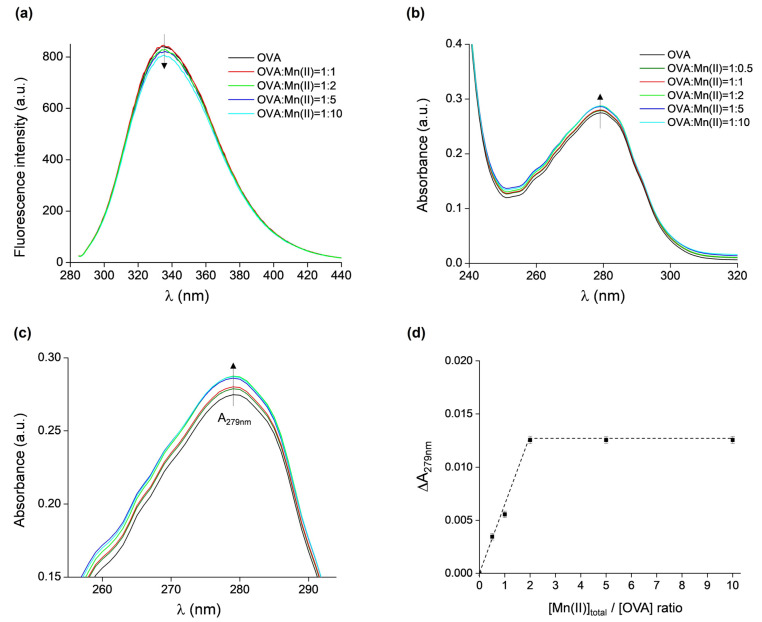
(**a**) Fluorescence emission spectra of OVA, excited at 280 nm, in the presence of increasing concentrations of Mn(II). (**b**) UV/vis absorption spectra of OVA in the presence of increasing concentrations of Mn(II). (**c**) Magnified part of UV/vis spectra shown in (**b**) (with the same color scheme as in (**b**)). (**d**) The difference in absorbance measured at 279 nm of OVA alone and of samples containing OVA in presence of Mn(II) vs. [Mn_tot_]/[OVA] ratios, ΔA_279nm_ = A_279nm_(OVA + *n*Mn) − A_279nm_(OVA), *n* = 0, 0.5, 1, 2, 5, 10.

The binding of Mn(II) to OVA was additionally investigated by fluorescence and UV/vis absorption spectroscopies. The intrinsic fluorescence of OVA is due to the presence of three tryptophan and ten tyrosine residues [3,4]. Fluorescence spectroscopy has been successfully used to investigate the interaction of OVA with various compounds, such as L-ascorbic acid, α-tocopherol, procyanidin B3, β-carotene, and astaxanthin [55], resveratrol [56], folic acid [57], Monascus pigment [58], caffeic acid [59], as well as metal-containing nanoparticles with gold and silver [60] or iron oxide [61]. The fluorescence emission spectra of OVA, excited at 280 nm, in the absence and presence of Mn(II) at protein:metal ratios of 1:1, 1:2, 1:5, and 1:10 are shown in Figure 5a. No fluorescence quenching is observed up to the 1:2 ratio, and insignificant quenching (ca. 5%) occurs for the 1:10 ratio. Moreover, there were no observable shifts in the spectra towards lower or higher wavelengths. This indicates that either the binding of Mn(II) to OVA does not induce protein conformational changes, which would in principle affect the local amino acid environment surrounding the fluorophores, or that the OVA binding sites for Mn(II) are not located in close proximity of the fluorophores.

The UV/vis spectra of the same samples (and additionally the protein:metal ratio of 1:0.5) showed only a minor increase in absorbance when measured at 279 nm (A_279nm_) with increasing Mn(II) concentrations (Figure 5b), confirming that the binding of Mn(II) does not affect the tertiary structure of OVA. However, the differences in A_279nm_ of OVA alone and of the OVA + Mn(II) samples are measurable (for clarity, a magnified part of Figure 5b is given in Figure 5c), and they were plotted as the function of [Mn_tot_]:[OVA] ratio (Figure 5d). It is observed that ΔA_279nm_ increased up to [Mn(II)_tot_]:[OVA] = 2, after which a plateau is reached. This result shows that a maximum of two Mn(II) ions can bind per one molecule of OVA, corroborating the findings from the EPR measurements.

## 3. Materials and Methods

### 3.1. Chemicals

Ovalbumin (OVA, purity ≥ 98%), bovine serum albumin (BSA, protease-free, fatty acid-free, globulin-free, purity ≥ 98%), human serum albumin (HSA, fatty acid-free, globulin-free, purity ≥ 99%), MnCl_2_·4H_2_O, and perchloric acid were purchased from Sigma-Aldrich, St. Louis, MO, USA. All samples were prepared using deionized water (Milli-Q, 18 MΩ∙cm, Merck KGaA, Darmstadt, Germany).

### 3.2. EPR Spectroscopy

The samples for EPR measurements contained 150 µL of 0.6 mM protein solution in deionized water, and 30 µL of the appropriate concentration of MnCl_2_ to obtain metal:protein concentration ratios of 0.2, 0.4, 0.5, 0.6, 0.8, 1, 2, 3, 4, 5, 7, and 10. All samples were incubated at room temperature for 1 h. MnCl_2_ solutions in deionized water in the concentration range of 0.1–6 mM were used for the Mn(II) calibration plot. A volume of 30 μL was drawn into a 1-millimeter-diameter gas-permeable Teflon tube (Zeus Industries Inc., Largo, FL, USA) and inserted into a quartz EPR cuvette (inner diameter 3 mm, Wilmad LabGlass, Vineland, NJ, USA) for spectra acquisition at room temperature. For the 77 K EPR experiments, 180 µL of samples containing Mn(II) and OVA in the metal:protein concentration ratio range of 0.2–10 were directly placed into the quartz EPR cuvette (inner diameter 3 mm, Wilmad LabGlass, Vineland, NJ, USA) and manually frozen in cold isopentane. 

All experiments were performed on a Bruker Biospin Elexsys II E540 EPR spectrometer. For the 77 K measurements, the liq.N2 dewar (WG-816-B-Q, Wilmad LabGlass, Vineland, NJ, USA) was used. The experimental parameters were as follows: For room-temperature measurements—microwave frequency 9.8 GHz, microwave power 10 mW, modulation amplitude 0.5 G, and modulation frequency 100 kHz. For 77 K measurements—microwave frequency 9.4 GHz, microwave power 1 mW, modulation amplitude 10 G, and modulation frequency 100 kHz.

### 3.3. Fluorescence Spectroscopy

The fluorescence emission spectra of OVA in deionized water, in the absence and presence of Mn(II) (at metal:protein ratios of 1:1, 2:1, 5:1, and 10:1), were recorded using the Agilent Cary Eclipse fluorescence spectrophotometer in the wavelength range of 285–450 nm. The excitation wavelength was 280 nm, the excitation and emission slit widths were 5 nm, and the scanning rate was 600 nm/min. 

### 3.4. UV/vis Spectrophotometry

The absorption spectra of OVA in deionized water, in the absence and presence of Mn(II) (at metal:protein ratios of 0.5:1, 1:1, 2:1, 5:1, and 10:1), were recorded on a UV/vis spectrophotometer (Lambda 35, Perkin Elmer, Waltham, MA, USA) in the wavelength range of 240−320 nm.

### 3.5. Data Fitting

The dissociation constants (K_d_) for Mn(II)/protein complexes were determined by fitting the data obtained from EPR measurements using a two-site binding model (Equation (1)):

y = B_max1_ · x/(k_1_ + x) + B_max2_ · x/(k_2_ + x), (1)

where y is the fraction of the occupied sites on the protein; x is the free Mn(II) concentration; B_max1_ and B_max2_ are maximal occupancies of binding sites 1 and 2; and k_1_ and k_2_ correspond to the appropriate dissociation constants.

## 4. Conclusions

The results from this study obtained from EPR measurements show that OVA contains two binding sites with different affinities for Mn(II); moreover, it was observed that OVA does not bind more than two Mn(II) ions per protein molecule, even in a 10-fold excess of Mn(II). This result was confirmed also by UV/vis spectroscopy. The binding of Mn(II) to OVA, or the presence of excess Mn(II), appears not to induce protein conformational changes, as evidenced by insignificant fluorescence quenching, ca. 5% for the concentration ratio OVA:Mn(II) = 1:10, and the lack of hypsochromic/bathochromic shifts. Moreover, it is likely that Mn(II) does not bind in close proximity to the fluorophores.

BSA and HSA exhibit different Mn(II) binding sites/modes compared to OVA, not displaying binding site saturation, which may be the result of their different physiological functions. It seems that OVA does not play a role in the transport and storage of manganese, however, it is likely involved in the regulation of manganese levels in vivo, possibly having a protective role from manganese overload.

The presence of the OVA-bound Mn(II) was confirmed by EPR spectroscopy at 77 K. The measured EPR hyperfine coupling constants suggest that Mn(II) is octahedrally coordinated to oxygen ligands; however, further competitive binding EPR studies at lower temperatures should be performed in order to elucidate the structural environments of the two Mn(II) binding sites on OVA. 

Although low-temperature EPR spectroscopy can detect protein-bound paramagnetic metal ions and further provide information on the symmetry and nature of the ligands, it is not optimal for metal ion quantitation due to the fact that double integration is hindered by background signals. Also, the signal intensity is not a good measure since it is sensitive to the nature of the ligands. The latter is also true for room-temperature EPR spectroscopy, however, it provides here an advantage in that the highest signal intensity is observed for the uncoordinated, free Mn(II)—namely [Mn(H_2_O)_6_]^2+^—while the protein-bound Mn(II) signal is broadened beyond detection. Therefore, the amount of the OVA-bound Mn(II) in this study was determined by measuring the amount of the unbound Mn(II) by EPR spectroscopy at room temperature. 

EPR spectroscopy has not frequently been used to investigate ligand binding since it detects only species that have at least one unpaired electron; however, when the ligand is, in fact, a paramagnet like Mn(II) investigated in this study, EPR is unrivaled. Namely, fluorescence and UV/vis absorption spectroscopies provide incredibly useful information regarding the changes in the protein environment caused by ligand binding, from which it is possible to calculate the total number of binding sites; however, the quantitation of free and bound paramagnetic ligands is possible only by EPR, which ultimately allows for discriminating between the numbers of binding sites with different affinities towards the ligand.

## Data Availability

All data are contained in this article and its Supplementary Materials.

## Supplementary Materials

The following supporting information can be downloaded at https://www.mdpi.com/article/10.3390/molecules29133221/s1. Figure S1: The calibration plot for MnCl_2_ in deionized water in the concentration range 0.1–6 mM; Figure S2: Room-temperature X-band EPR spectra of 0.5 mM OVA in deionized water, pH 5.5, incubated with MnCl_2_ at different OVA:Mn(II) concentration ratios: (**a**) 1:0.2 (black), 1:0.4 (red), 1:0.6 (blue), 1:0.8 (green), 1:1 (purple), and (**b**) 1:1 (black), 1:2 (red), 1:3 (blue), 1:5 (green), 1:7 (purple), 1:10 (yellow).
