# Supplementary material for: The Role of Ovalbumin in Manganese Homeostasis during Chick Embryogenesis: An EPR Spectroscopic Study
Source: Molecules. 2024 Jul 7;29(13):3221. doi: 10.3390/molecules29133221 (PMC11243369; doi:10.3390/molecules29133221)
Supplement: Supplementary file 1 [file molecules-29-03221-s001.zip › molecules-3070644-supplementary.pdf]

Supplementary Material

for

Ovalbumin may play a role in the protection from manganese toxicity during chick embryogenesis: an EPR spectroscopic study

Ana Vesković[1], *Aleksandra M. Bondžić* [2], *Ana Popović Bijelić*[1*]

[1]*Faculty of Physical Chemistry, University of Belgrade, Studentski trg 12-16, 11158 Belgrade, Serbia*

[2]*Vinča Institute of Nuclear Sciences, National Institute of the Republic of Serbia, University of Belgrade, P.O. Box 522, 11000 Belgrade, Serbia*

[*]Corresponding author: Ana Popović Bijelić, ana@ffh.bg.ac.rs; Faculty of Physical Chemistry, University of Belgrade, Studentski trg 12-16, 11158 Belgrade, Serbia; Phone/Fax. +381(0)112187133.

List of Figures:

Figure S1. The calibration plot for $MnCl_2$ in deionized water in the concentration range 0.1–6 mM.

Figure S2. Room-temperature X-band EPR spectra of 0.5 mM OVA in deionized water, pH 5.5, incubated with $MnCl_2$ at different OVA:Mn(II) concentration ratios: (**a**) 1:0.2 (black), 1:0.4 (red), 1:0.6 (blue), 1:0.8 (green), 1:1 (purple), and (**b**) 1:1 (black), 1:2 (red), 1:3 (blue), 1:5 (green), 1:7 (purple), 1:10 (yellow).

1. Mn(II) calibration plot

Figure S1 shows the calibration plot used for the determination of the unbound Mn(II) concentration in OVA/Mn(II) samples. The graph shows a linear fit (through zero) of the data points obtained from room temperature EPR spectra of 0.15, 0.75, 1.5, 3.0, 4.5 and 6.0 mM $MnCl_2$ in deionized water. The normalized intensity of the first line in the EPR spectra was plotted as the function of Mn(II) concentration.

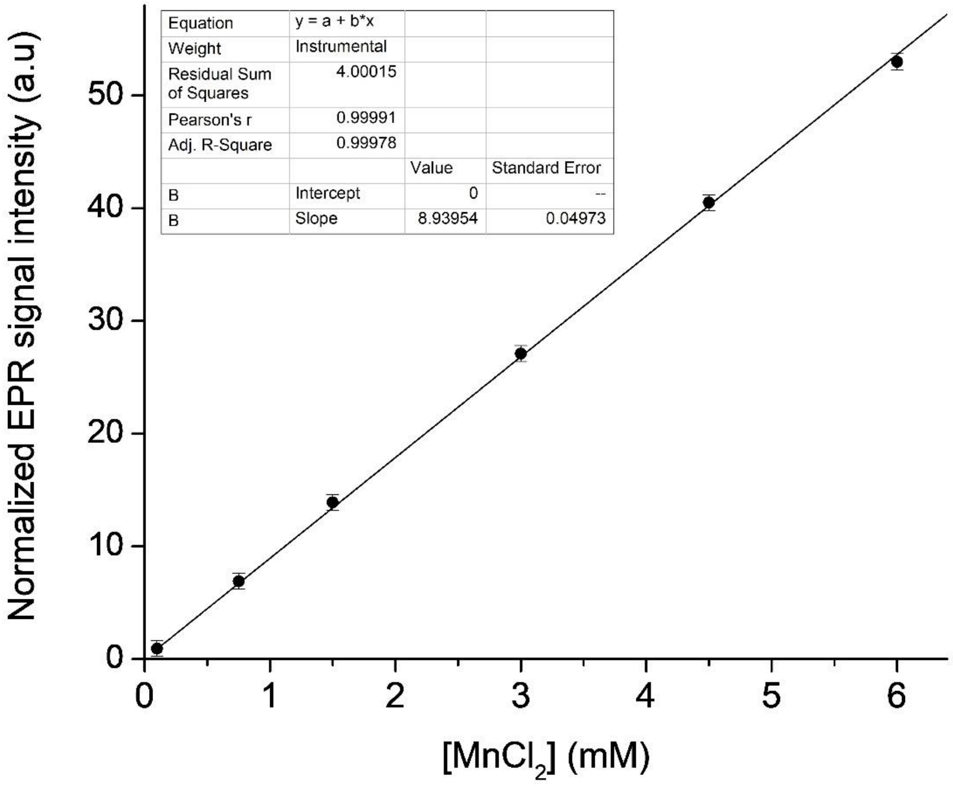

Figure S1. The calibration plot for $MnCl_2$ in deionized water in the concentration range 0.1–6 mM.

2. Room temperature X-band EPR spectra of Mn(II)/OVA samples

Figure S2 shows the room temperature EPR spectra of samples containing Mn(II)/OVA in the concentration ratios 0.2, 0.4, 0.5, 0.6, 0.8, 1, 2, 3, 4, 5, 7, and 10. The concentration of OVA was kept constant (0.5 mM) in all samples, while the concentration of Mn(II) was varied accordingly.

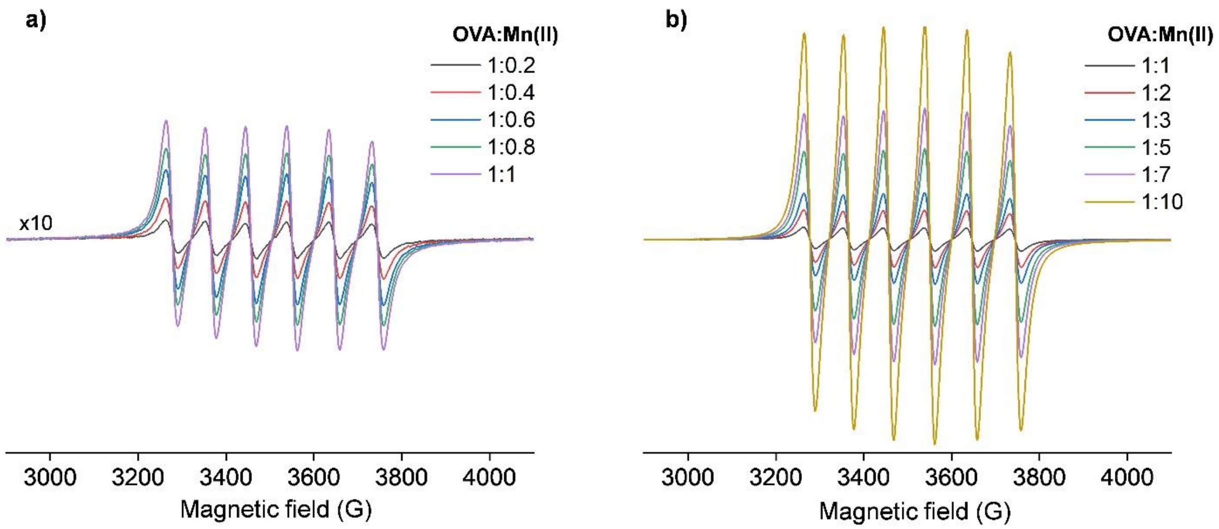

Figure S2. Room-temperature X-band EPR spectra of 0.5 mM OVA in deionized water, pH 5.5, incubated with $MnCl_2$ at different OVA:Mn(II) ratios: (**a**) 1:0.2 (black), 1:0.4 (red), 1:0.6 (blue), 1:0.8 (green), 1:1 (purple), and (**b**) 1:1 (black), 1:2 (red), 1:3 (blue), 1:5 (green), 1:7 (purple), 1:10 (yellow). The spectra in (**a**) and (**b**) are shown on different scales for clarity of observation, the scale in (**a**) is magnified 10x. Note that the EPR spectrum for the OVA:Mn(II)=1:1 ratio is shown in both, (**a**) and (**b**), for comparison.
